# A Micro-Computed Tomographic Evaluation of Root Canal Morphology of Mandibular First Molars in a Black South African Subpopulation

**DOI:** 10.3390/jcm14072301

**Published:** 2025-03-27

**Authors:** Casper Hendrik Jonker, Federico Foschi, Ericka Noelle L’Abbé, Anna Catherina Oettlé

**Affiliations:** 1 Faculty of Health, Peninsula Dental School, University of Plymouth Ground, Plymouth PL6 8BT, UK; 2 Truro Dental Education Facility, Knowledge Spa, Royal Cornwall Hospital, Truro TR1 3HD, UK; 3 Eastman Dental Institute, Rockefeller Building, 21 University Street, London WC1E 6DGL, UK; f.foschi@ucl.ac.uk; 4 Forensic Anthropology Research Centre, Department of Anatomy, Faculty of Health Sciences, University of Pretoria, Pretoia 0031, South Africa; ericka.labbe@up.ac.za; 5 Anatomy and Histology Department, School of Medicine, Sefako Makgatho Health Sciences University, Pretoria 0204, South Africa; profoettle@gmail.com

**Keywords:** African continental ancestry group, dental pulp, microcomputed tomography, root canals, South Africa

## Abstract

**Background**: This study evaluates the root canal anatomy (main and accessory canals) of mandibular first molars in Black South Africans using micro-computed tomography. The effect of sex, arch side, and age are also investigated. **Methods**: The number of root canals in each tooth and individual roots (including an observation of the middle-mesial and middle-distal canals), the number of accessory canals, accessory canal type, root canal thirds, and the prevalence of apical deltas were recorded. A chi-squared test of association (*p* < 0.05) was used to report on variables (sex, arch sides, and age). **Results**: In most teeth, either three (55.81%) or four canalled (24.42%) configurations were present. The middle-mesial canal was found in 18.6% of teeth (males and females), with a higher prevalence among females (23.68%). The middle-distal canal was found in 3.49% of teeth. Chamber canals were identified in 4.7% of teeth. Accessory canals most likely located in the apical third were found in approximately 84.9% and 86.1% of mesial roots and distal roots, respectively. Apical deltas were identified in 15.12% of mesial and 20.93% of distal roots. **Conclusion**: Root canal anatomy in this population is diverse and includes differences in arch sides between the sexes.

## 1. Introduction

Root canal treatment is considered a viable option to retain a tooth. The procedure includes a series of steps to remove infected organic material with the invading micro-organisms from the root canal system [1]. The root canal space often comprises a complex network of root canals, and challenging pulpal anatomy has been identified as a possible reason for treatment failures [1,2]. Mandibular first molars generally contain three or four canals [2,3,4]. According to the literature, variants exist in the number of canals between population groups [2,3,5]. The mesial (M) and distal (D) roots may contain third canals known as the middle-mesial (MM) and middle-distal (MD) canals, respectively [6]. Teeth with one, two, six, seven, eight, and 11 canals have also been reported [2].

According to Ahmed et al., accessory canals can be described as small canals that leave the main canal and usually, but not always, communicate with the external root surface or furcation area. They can be located anywhere on the root and can be patent, blind, or looped [7]. Multiple accessory canals (ACs) can be present in either the coronal, middle, or apical thirds [8], while main root canals often terminate in apical deltas with multiple portals of exit at the apex [7].

Micro-computed tomography (micro-CT) has emerged as an appropriate diagnostic tool to explore the internal morphology of teeth in a non-invasive and accurate manner [9]. Nielsen and his co-workers were some of the earliest researchers to report their findings in a maxillary first molar using this modality [10]. Since then, others have used it to report on root canal morphology of mandibular first molars [9]; however, currently, there is a deficiency in data related to micro-CT studies on individuals of known age and sex within populations. No previous research could be found in the literature using micro-CT to evaluate root canal anatomy in a South African population. The aim of this study is to describe, for the first time, the main and accessory root canal anatomy of mandibular first molars and their variants in Black South Africans using micro-CT.

## 2. Materials and Methods

### 2.1. Sampling Method and Approval

**Figure 1:**
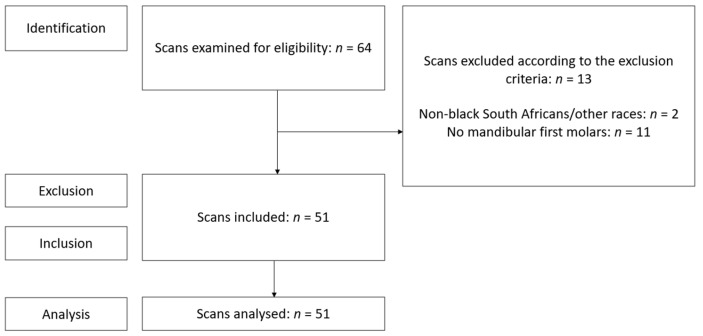
A STROBE flow diagram depicting scan eligibility according to the exclusion and inclusion criteria.

Permission to conduct the study was obtained from the Research Ethics Committee (Protocol number: 298/2020). The study design was quantitative, descriptive, cross-sectional, and observational. The Strengthening the reporting of observational studies in epidemiology (STROBE) guidelines were considered in this study (Figure 1) [11].

Human skulls with known age, sex, and population affinity were sourced from human bone collections held in secured facilities [12]. Family members gave permission for research purposes where donations were made and formed part of the whole-body donation programme. Samples derived from unclaimed bodies are protected by the National Health Act 61 of 2003 [13]. Individuals were identified as Black South Africans at the time of death, and age and sex were recorded.

### 2.2. Sample Selection

A total of 64 possibly suitable micro-CT scans were identified from a convenience sample, and all available scans were considered (Figure 1). A total of 86 mandibular first molars from 51 scans were deemed eligible according to the inclusion and exclusion criteria. The sample contained teeth from both arch sides (left and right), from males and females of different ages.

### 2.3. Inclusion Criteria

The following inclusion criteria were followed: scans of adequate quality for proper segmentation and isolation of pulps (no blurring or double visions); the presence of at least one mandibular first permanent tooth; teeth with intact, completely formed roots; and individuals identified as Black South Africans of known age.

### 2.4. Exclusion Criteria

Teeth with incomplete root formation (immature roots), root fractures, apical root resorption, previous root canal treatments, teeth filled with metallic restorations, root fractures, severe decay into root canals, root resorption into root canal space, teeth from individuals from other populations or where age was not known were discarded. Faulty scans of inadequate quality or resolution were also not considered.

### 2.5. Micro-CT Scanning Parameters

The complete maxillae or mandibulae of available skulls were scanned at Necsa (South African Nuclear Energy Corporation, Pelindaba, South Africa) using the Nikon XTH 225L industrial CT system (Nikon Metrology, Leuven, Belgium) with the following parameters: 100 kV voltage, 100 mA current, and 2.00 s exposition time per projection. The x-ray spot size ranged between 0.001 and 0.003 mm (1–3 μm), with a pixel size of 200 μm × 200 μm. The scanning procedure generated two-dimensional projection images which were exported to the embedded software, while the final volumes were constructed with the Nikon CT Pro version 4.4.3 software (Nikon Metrology, Grand River Brighton, MI, USA). The isotropic voxel size ranged from 62.4 to 74.2 µm. For optimal resolution, the sample was zoomed in to the maximum magnification possible, resulting in the minimum effective pixel size, while still ensuring that the entire part remained within the image window throughout the scan. More magnification would, therefore, be possible for smaller mandibles, resulting in a scan with smaller voxels. The final three-dimensional (3D) volumes were imported into the Avizo 2019 visualisation software (Thermo Fisher Scientific Inc., Waltham, MA, USA) for the subsequent post-acquisition processes [14].

### 2.6. Segmentation, Alignment, and Image Acquisition

Firstly, 3D rendering of the object was performed on all micro-CT scans by isosurface determination in Avizo. Each molar was virtually extracted by cropping and through a segmentation process. A continuous line of sliding landmarks was placed along the cemento–enamel junction (CEJ)—a line that is always present and repeatable on each tooth in the mandibular arch [15]. The global axis of each virtual mandible was then aligned using a grid. The same process was followed for each scan to align images precisely and, therefore, reduce possible bias and oblique sections. With the use of Avizo, a best-fit plane was created by connecting all the sliding landmarks. This plane also served as a reference to re-align the micro-CT image stacks and as a tool (when moved in an apical or coronal direction) to compare teeth in different arches. A region-based automatic segmentation procedure, known as the watershed, was carried out to allow for virtual extraction and 3D observation of each tooth component [16]. Segmentation is a process where a tooth is virtually isolated from the jaw using different pieces of software, with the enamel, dentine, and pulp labelled following exact parameters. On completion, the pulp of each tooth was isolated and extracted, rotated, and magnified, if necessary, to identify any hidden root canal anatomy. The brightness, contrast, and sharpness of each scan were adjusted within the parameters of Avizo for optimal visualisation and to distinguish the enamel, dentine, and pulp. The same settings were used for each tooth to ensure a calibrated experience. Masking or multiple-slice editing, a selective editing procedure, was used to remove dentinal cracks and eliminate any communication between the pulpal space and the external tooth surface [17].

### 2.7. Analysis of Scans

**Figure 2:**
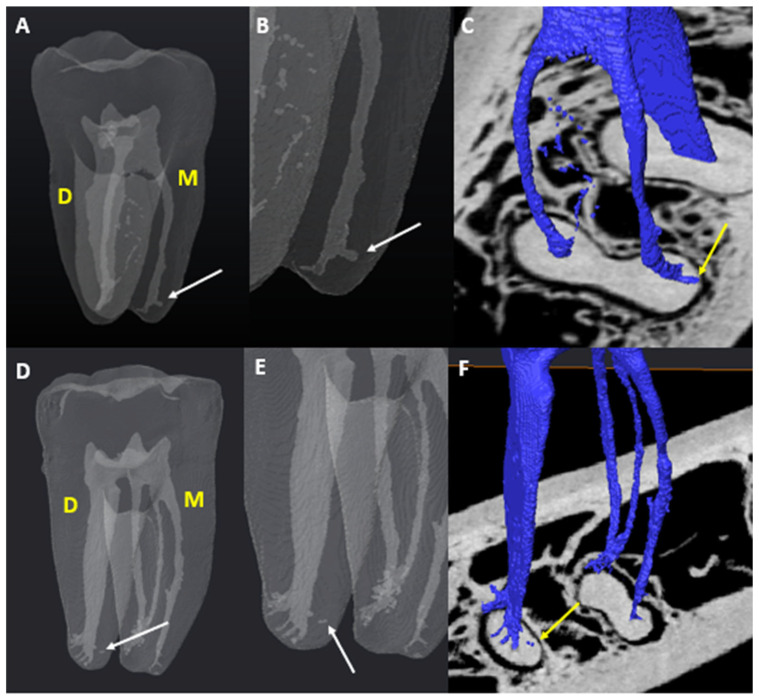
Micro-CT illustrations of ACs found in M and D roots: (**A**) patent AC in the M root (white arrow); (**B**) patent AC under a magnified view; (**C**) pulp–root surface interface view of a patent AC (yellow arrow); (**D**) blind-ended AC (white arrow); (**E**) blind-ended AC under a magnified view (white arrow); (**F**) pulp–root surface interface view of a blind-ended AC (yellow arrow).

**Figure 3:**
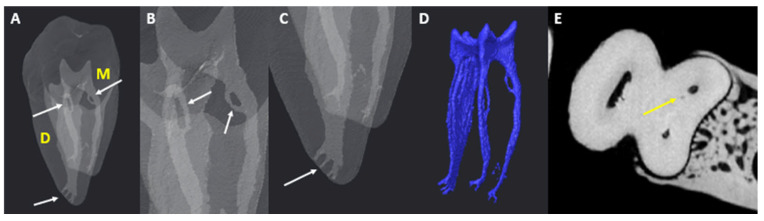
Micro-CT illustrations of looped ACs and apical delta: (**A**) looped AC (white arrows); (**B**) looped AC under a magnified view (white arrows); (**C**) apical delta (white arrow); (**D**) pulpal view of the isolated root canal complex; (**E**) cross-sectional view of the root canals in the blind-ended AC under a magnified view yellow arrow).

The observation of scans followed a sequence similar to that outlined by Jonker et al. [18]. Data collection was first completed by the main researcher, who has experience in endodontics and 3D/micro-CT imaging, including the use of Avizo. To reduce the risk of bias, patient identifiers were removed, and unique codes were allocated to each scan without any prior knowledge of sex, arch side, or age. The same settings and parameters within Avizo for optimal contrast and brightness were used for each scan. The number of main canals in each tooth and individual roots was calculated, including the MM and MD canals and any variants that were present. To report on ACs, the guidelines reported by Ahmed et al. [7] regarding ACs were followed during the observations. According to Ahmed [17], one specific area of potential ambiguity between studies involves the distinction between apical AC (accessory canal) and apical bifurcation. They advocate for clear descriptions to increase comparability of results. For this reason, the guidelines and illustrations of Xu et al. [19,20,21] and Ahmed [17] were considered in this study. Root canal bifurcation is identified as the main root canal dividing into two separate branches of similar size and diameter, each with a separate exit on the apical surface. Figure 2 and Figure 3 show the anatomy of accessory root canals.

A second researcher (a Specialist and Consultant in prosthodontics with endodontic experience) was approached to assist with interobserver reliability. Both researchers were calibrated by observing and analysing micro-CT images and discussing root canal anatomy on two randomly chosen scans unrelated to the reliability test sample of images. The reliability test sample included micro-CT images of approximately 20% of the total sample of teeth (*n* = 17/86) which were randomly selected. Each researcher recorded their results independently and the results were later compared. The findings of both researchers were recorded in Microsoft Excel 2016 (Microsoft Corporation, Redmond, WA, USA). Results were accepted in the event of agreement, and any discrepancies were discussed until a consensus was reached. For example, in cases where ambiguous opinions were present relating to the different types of accessory canals (blind-ended vs. looped vs. patent), the issues were resolved by evaluating the trajectory, patency, exit on the external root surface, and connection to the main root canal system of each AC. Intrarater reliability was also determined by repeating the observation on the same selection of scans within one week [22].

### 2.8. Statistical Analysis

The R Statistical Software, version 4.1.1 (R Core Team 2021.R, Vienna, Austria), was used to perform statistical analysis. The variables in the study (sex and arch sides) were analysed using a chi-squared test of association (*p* < 0.05). The effect of age was also analysed using a chi-squared test of association (*p* < 0.05), and suitable teeth were divided into the following age groups: 20–30, 31–45, and 46+ years of age. To determine intra- and interobserver reliability, the unweighted Cohen’s Kappa coefficient test was used (*p* < 0.001).

## 3. Results

### 3.1. Description of the Sample and Examiner Agreement

A total of 86 molars from 52 individuals (males and females) were included. The sample contained more male teeth (*n* = 48/86, 55.81%) than female teeth (*n* = 38/86, 44.19%). The distribution between left (L) and right (R) sides was relatively even, though there were slightly more teeth from the right side (*n* = 44/86, 51.16%) than from the left (*n* = 42/86, 48.84%). Intra- and interrater agreement was 98.8% and 97.7%, respectively.

### 3.2. Total Number of Canals

**Figure 4:**
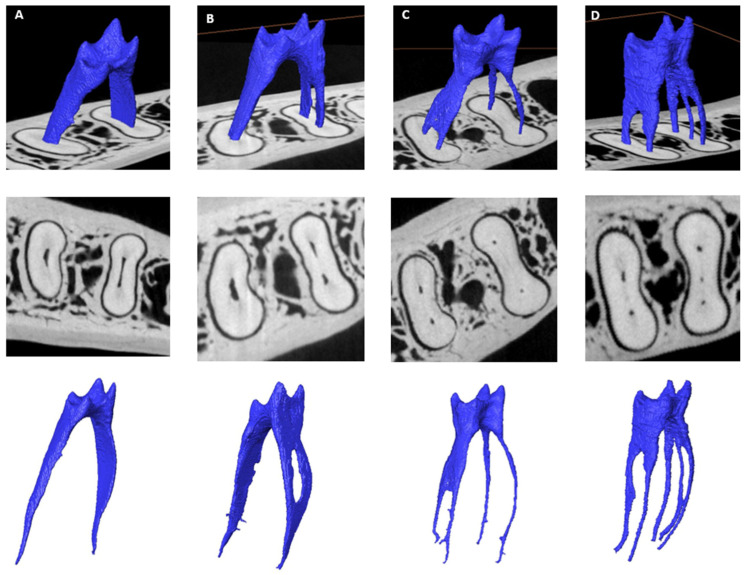
Micro-CT illustrations on the number of root canals found in mandibular first molars: (**A**) mandibular first molar with two canals displayed as combined pulp and cross-section, cross-section alone, and isolated pulp (top to bottom); (**B**) three main canals; (**C**) four main canals; (**D**) five main canals.

The number of canals and their variants (prevalence between males and females and between L and R sides) are summarised in Table 1. In most teeth, either three or four canals were present. A fifth canal was found in approximately 12.8% (*n* = 11/86) of teeth. The mean number of canals in two-rooted teeth was 3.44 (standard deviation: 0.81; median: 3.00; range: 2.00 and 5.00) and 3.50 (standard deviation: 0.71; median: 3.50; range: 3.00 and 4.00) for the three-rooted ones. In individual roots, the mean number of canals in the M root of two-rooted teeth was 2.12 (standard deviation: 0.50; median: 2.00; range: 1.00 and 3.00) and 1.32 (standard deviation: 0.54; median: 1.00; range: 1.00 and 3.00) in the D root. In three-rooted teeth, the mean number of canals was 2.00 (standard deviation: 0.00; median: 2.00; range: 2.00 and 2.00) in the M root and 1.50 (standard deviation: 0.71; median: 1.50; range: 1.00 and 2.00). Typical root canal configurations found in this study are depicted in Figure 4. The presence of two and four canals was common in females, while more males than females had three canals. Teeth with two canals were common on the L side. In contrast, molars with three canals were common on the R side.

### 3.3. Number of Canals per Root

**Figure 5:**
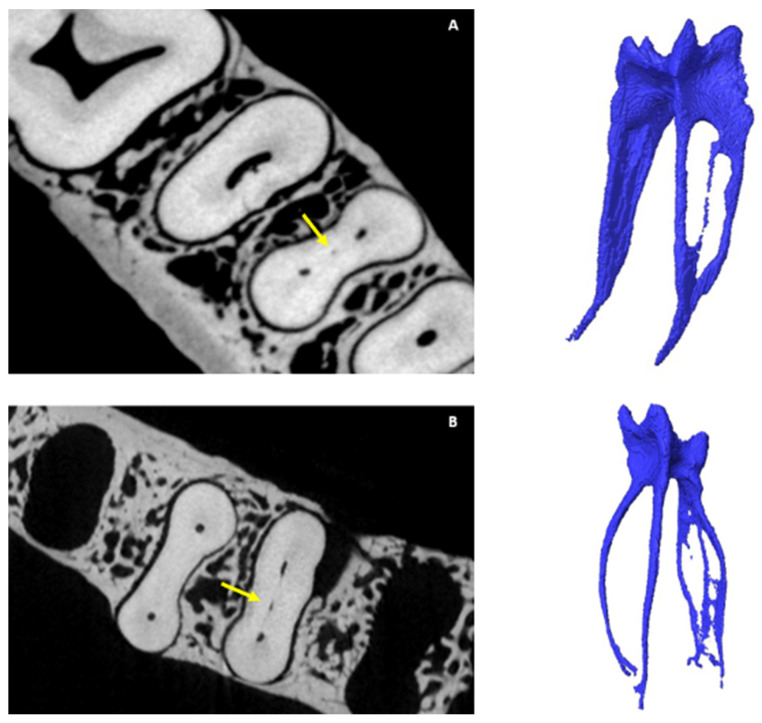
Micro-CT illustrations on additional canals found in the M and D roots of mandibular first molars: (**A**) cross-section and isolated pulp of the mid-mesial (MM) canal (yellow arrow); (**B**) mid-distal (MD) canal.

Findings in different roots are summarised in Table 2. Most M roots had two canals and were commonly found in males. A single canal in the M root was present in 6.98% of teeth and was common in females. The MM canal was found in 18.6% of the mandibular samples and was more frequent in females than males, as well as being more often found on the R side. A single canal was more commonly found in the D root. Two canals in the D root were more common in females than in males. The MD canal was found in 3.49% (*n* = 3/86) of teeth. Single canals in the mesial root were most often found on the L side. The MD canal was only found on the L side. Figure 5 depicts some of the MM and MD configurations identified in this study. Two molars with additional distolingual roots were identified (*n* = 2/86, 2.3%); in one, a single canal was identified in the additional root, and none in the other.

### 3.4. Chamber Canals

**Figure 6:**
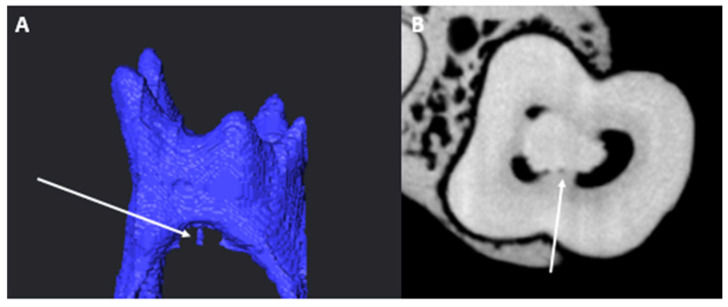
(**A**) Micro-CT illustrations of a blind-ended CC (white arrow); (**B**) cross-sectional view of the same CC (white arrow).

Chamber canals (CCs) were identified in a small number of teeth (*n* = 4/86, 4.7%). No looped or patent CCs were found, only blind-ended ones. More female molars had CCs (*n* = 3/86, 3.5%) than male molars (*n* = 1/86, 1.2%). Findings between the L and R sides were similar (two each). Figure 6 illustrates a typical blind-ended CC found in the sample.

### 3.5. Accessory Canals (ACs)

A total of 204 ACs (excluding deltas) were identified from the molar samples. The mean average number of ACs was 2.37 (standard deviation: 1.76; median: 2; range: 0.00–6.00).

#### 3.5.1. M Root

Findings are summarised in Table 3. Most of the mesial (M) roots contained ACs (*n* = 65/86, 75.58%). The prevalence was found to be higher in males. A predominance of the apical third was noted for both sexes and sides. All types of ACs were identified, but the patent type was more common. The male samples contained more blind-ended ACs and apical deltas. The mean number of ACs for the M root was 1.31 (standard deviation: 1.25; median: 1.00; range: 0.00–6.00). More ACs were noted on the left side.

#### 3.5.2. D Root

Findings are summarised in Table 4. ACs were present in most distal (D) roots, although they were more common in males. A predominance of the apical third was noted. Patent ACs were the most common across both sexes, although all types were observed. Apical deltas were more common in males and on the L side, although the overall prevalence was 20.93%. More ACs (excluding apical deltas) were found in males. The total number of ACs in the D root was 91 (mean: 1.06; standard deviation: 1.07; median: 1.00; range: 0.00–4.00), and they were more commonly found on the L side. Two molars had additional distolingual roots (2/86, 2.3%), but no ACs were observed in the extra roots.

### 3.6. Effect of Arch Sides, Sex, and Age

A chi-squared test of association revealed no statistically significant variation in the main canals by sex (*p* = 0.631) or by side (*p* = 0.625). No statistically significant variation was noted in root canals from the M root in terms of sex (*p* = 0.051) or side (*p* = 0.167), nor in the D root in terms of sex (*p* = 0.777) or side (*p* = 0.194). In the M root, no statistically significant relationship between sexes and sides in the root regions where ACs (including apical deltas) were located was noted (sex: *p* = 0.176; arch side: *p* = 0.339). No sex- or arch-side-driven relationships were observed in relation to the type of AC (sex: *p* = 0.430; arch side: *p* = 0.923) and number of ACs (sex: *p* = 0.631; arch side: *p* = 0.626). Similarly, in the D root, no statistically significant relationship was noted between sexes and sides, on one hand, and root regions, on the other (sex: *p* = 0.152; arch side: *p* = 0.928). No sex- or arch-side-driven relationships were found in relation to the type of AC (sex: *p* = 0.185; arch side: *p* = 0.880) and number of ACs (sex: *p* = 0.693; arch side: *p* = 0.318). With regard to age groups, no statistically significant relationship was noted in relation to the number of canals in the total number of teeth, including their individual roots (all teeth: *p* = 0.950; M root: *p* = 0.582; D root: *p* = 0.429). There was also no statistically significant relationship between age groups and the presence of ACs in root thirds, AC types, or the number of ACs in the M root (*p* = 0.659, *p* = 0.945, *p* = 0.950, respectively) and the D root (*p* = 0.406, *p* = 0.615, *p* = 0.431, respectively).

## 4. Discussion

A typical mandibular first molar has three or four canals [1], but the prevalence might differ across populations. Similar observations were recorded in a Malaysian population, where three canals were found in 53.3% of individuals and four were found in 22.2% of the population [23]. In this particular Malaysian study, 23.3% of teeth had two canals, a percentage that is higher than the 6.98% of teeth with two canals found in this study. With respect to teeth with two canals, researchers may have a degree of subjectivity in their viewpoints. Interpretation of the literature has led to the conclusion that there is no clear distinction between two separate canals joined by a sheet-like isthmus from coronal to apical or fan-type I configuration [24] and a single flat or ribbon-shaped canal [25]. The appearances are similar, a fact that may explain variations across reports.

In addition, other factors including population differences, genetics, external factors, or geographic locations cannot be excluded. In this study, the presence of a fifth canal was noted in roughly 12.8% of teeth, a percentage that is higher than that found in a United Arab Emirates (UAE) study, where a fifth canal was present in 6.9% of molars [26]. In the UAE study, CBCT was used, and although suitable for reporting on pulpal morphology, CBCT has limited potential to accurately identify fine details [17]. It is, therefore, possible that small additional canals remained undetected.

The number of canals also varied between roots. A single canal in the M root was noted in 6.98% of Black South Africans. Also, more female molars had a single canal in the M root (approximately 13.2%) than male molars (approximately 2.1%). In a recent South African CBCT study involving a mixed population (Whites, Indians, Blacks, and other groups), Tredoux et al. (2020) [6] reported a lower prevalence of 0.5% of single canals in the M root [6]. Interestingly, in a Tanzanian study using extracted teeth from individuals of African descent, and using a clearing and staining technique, no single-canalled M roots were noted [27]. Two canals in the M root were found in approximately 72.1% of the sample of Black South Africans. Among Ugandans, a lower prevalence of 61.6% [28] was noted. In contrast, in a Tanzanian population, all M roots had two canals [27].

The MM canal is located between the main mesiobuccal (MB) and mesiolingual (ML) canals. Locating these canals can be challenging for clinicians, as their orifices are often covered by dentine. A recent micro-CT investigation determined that 7.5% of MM canals were located 2 mm further apically than the cemento–enamel junction (CEJ) [29]. Efforts to gain access may lead to perforations, and treating clinicians should be mindful of their presence and location. Specialised equipment and high magnification might be required to locate canals with relative safety [29]. A prevalence of 18.6% was found among Black South Africans in the current investigation, similar to the findings of Tredoux et al. (2021) [6] (20%). In a micro-CT investigation, Versiani et al. (2016) [30] reported a prevalence of 22.1% among Brazilians.

Variations were also noted in the D root. In this study, one D canal was noted in roughly 70.9% of teeth, a percentage that is similar to that obtained by a Brazilian micro-CT study where a prevalence of 76% was noted [31]. In a recent worldwide CBCT investigation involving 23 nations from five continents, different findings were reported, although some nations reported a similar prevalence value to that found in this study: Belgium (71.2%), India (70.4%), as well as Costa Rica and Mexico (71.6%) [32]. In this worldwide study, the reported prevalence among South Africans was 60%, which is lower than the value from this study. The South African sample was derived from a mixture of populations: Asians (Indian origin), Whites, and a minority of indigenous Africans; indigenous Africans were the minority. The distribution of each population group was not specified in this worldwide study. However, a comparison between this study and the worldwide investigation is still valuable, as CBCT technology is considered suitable for the identification of main canals [6]. The presence of two D canals was noted in approximately 25.6% of teeth in this study, a finding similar to that from a Portuguese study that included western Europeans and Chinese Asians (29.2% and 20.5%, respectively) [33]. In the Tanzanian study, the prevalence of two canals was higher (40.4%) [27].

The prevalence of the MD canal has been established to be as high as 8% [31]. In the present study, a prevalence of 3.49% for the MD canal was noted. In African studies (Uganda [28], Tanzania [27]), no additional D canals were found. It is important to note that clearing and staining techniques have been shown to have certain limitations. Authors have stated that the flow of dyes to all areas cannot be guaranteed, especially in teeth with complex root canal configurations and where debris or vital tissues are present [34]. On the other hand, Tredoux et al. (2021) reported a prevalence of 7% for MD canals in mixed South African populations [6].

However, micro-CT reports on the D root are rare. A micro-CT study from the United States of America reported a prevalence of MD canals of 11% [35]. In this study, two radix entomolaris (RE) teeth were identified (2.3%), in agreement with findings from Brazilian populations (2.6%) [36]. An RE is a mandibular molar where an additional root is present on the distolingual side, a phenomenon which can have significant clinical implications. Careful investigation and planning are required to identify any additional roots or canals in these teeth [37]. The use of CBCT might also be beneficial to clinicians to accurately identify complex root morphology.

Treating clinicians should be aware that accessory root canal morphology may have clinical implications. Accessory root canals are potential communication pathways between the pulp and the external environment. According to the descriptors and visual illustrations by Ahmed et al. [7,17], they may exhibit patency (a clear communication pathway between the main root canal and the external root surface), be blind (interrupted root canal pathway en route to the exit on the external root surface), or be looped (a semi-lunar-type root canal pathway where both ends are connected to the main root canal). Accessory pathways are formed when blood vessels are encapsulated during root development and exist as interrupted developmental areas of the Hertwig root sheet (HERS) [20]. Infected ACs can contain infected organic tissues with micro-organisms. Causative micro-organisms can move freely between the site of infection and the periodontal ligament space, causing periodontal diseases. A challenge is the fact that AC anatomy may remain out of reach of traditional root canal instruments and irrigation solutions [38]. This challenge can be problematic, with any remaining micro-organisms (and their byproducts) and irreversibly inflamed or necrotic organic tissue possibly having a catastrophic effect on the treatment outcome. A patient could experience continuous symptoms after treatment, leading to periapical pathology and treatment failure. On the other hand, periodontal disease with accompanying micro-organisms can gain access to the root canal system through accessory portals [39].

Chamber canals may also serve as communication portals between the periodontium in the furcation region and the root canal system [40]. Similar to other types of ACs, they can be patent, blind, and looped in nature [7]. In the present study, the prevalence of blind-ended CCs was found to be 4.7%. A similar prevalence (5.1%) was reported in a recent micro-CT study of German and Egyptian individuals, referred to as diverticulae [40]. No patent or looped ACs were found in this study, whereas a prevalence of 4.2% for patent (inter-radicular) ACs was noted among Germans and Egyptians.

In the current investigation, the overall prevalence of ACs was 75.58%. This value is lower than that obtained in a recent Lithuanian study (85%) [41]. As in this study, a higher prevalence was noted in the M root, with a predominance of the apical region in the Lithuanian and other studies [8,20,35,42]. The present study sample contained a few radix entomolaris teeth (a mandibular molar with an additional root on the distolingual side) [37]. According to the literature, the main D root or additional roots may contain several ACs [8,42], a phenomenon which is contradictory to our current findings, as no ACs were observed in the additional roots in the study sample. In this study, the M root had, on average, 1.31 ACs, while the D root had an average of 1.06. These findings are similar to those from a Burmese (Mayanmar) study, where the M root contained, on average, 1.63 ACs, ranging between 0 and 6 [43]. Interestingly, the current investigation found no statistically significant relationship among arch side, sex, or age groups.

It has been determined that ACs are predominantly present in the apical 3 mm of roots. Where apical surgery is required, a minimum of 3 mm must be removed during apical root resection [35]. The findings of this study agree with other findings, and special consideration must be given in cases in which apical surgery is required. Apical deltas were also commonly found in this investigation. A higher prevalence was noted in the D root than in the M root (13.95% and 19.77%, respectively). A similar observation was made by Gu et al., investigating a Chinese population using micro-CT [42]. A Turkish micro-CT study, on the other hand, noted a prevalence of 13.01% for apical deltas in the M root, a value which is similar to that obtained in this investigation [29].

As micro-CT reports are limited, root canals, perhaps missed by other techniques, would not be universally present. Future studies utilising micro-CT on other populations could verify the findings of this study and establish whether features missed by other techniques (CBCT or clearing and staining) can be identified by micro-CT. Furthermore, investigation of the current literature led to the conclusion that expansion on the current terminology might be required, in particular the precise description of blind-ended accessory canals alongside a clear distinction between bifurcating canals and patent accessory canals in the apical third [17].

The small sample size in this study can be considered a limitation. A convenience sample was used, and all available scans were evaluated to source teeth. Potential biases in sample selection were reduced to a minimum. All available skeletal remains were considered for scanning purposes irrespective of age, socio-economic status, or background. The authors acknowledge that a convenience sample might limit the demographic and geographic representation of the broader Black South African population. However, the sample size of this study includes a fair distribution of males (*n* = 48/86, 55.81%), females (*n* = 38/86, 44.19%), arch sides (right side: *n* = 44/86, 51.16%; left side: *n* = 42/86, 48.84%), and ages (ranging between 20 and 90 years), such a distribution being sufficient to conduct statistical analysis. According to a power calculation, in order to detect a medium effect size and achieve a power of 0.7–0.8, a sample size of between 86 and 108 data points is sufficient for most comparisons, as was the case for the current study (*n* = 86) [44]. Importantly, the current study constitutes novel exploratory work on these individuals. Similar sample sizes have been used by other authors [2,17].

Other limitations include the fact that the current investigation only focussed on one population group within South Africa and that a dried skeletal collection was used. Some minor dentinal cracks noted in dried specimens could have been missed during multiple slice editing, resulting in cracks not being masked and, perhaps, being mistaken for an accessory canal. However, the advantage of using a software-based segmentation process to enhance accuracy during sample selection and data collection was that it allowed for precise comparisons of root canal anatomy between the left and right arches, between sexes, and across different age groups. First molars could be selected based on their morphology, alignment, position in the arch, and relationship to adjacent teeth (if present), enabling the reliable attribution of teeth to specific individuals. The authors also accept that a degree of subjectivity may have been present during the identification and calculation of root canal anatomy, including accessory canals. Finally, the larger isotropic voxel sizes/resolution of scans (62.4 µm–74.2 µm) used in this study could also be considered a limitation. Bai et al. [45] suggested that diagnostic accuracy for detecting ACs may increase with voxel sizes between 75 µm and 80 µm. Considering this, the voxel size range of the current study is below the suggested range for an increased possibility of AC detection. In addition, during the calibration process, images of scans were evaluated in-depth to ensure an accurate detection and identification of all types of ACs by both researchers. Images were viewed from different angles, different colours were allocated to tooth structures, the pulp–root surface interface view within the Avizo software was adjusted to promote accurate visualisation, and images were magnified and enlarged. As a result, excellent intra-and inter-reliability scores were achieved (intra- and interrater agreement: 98.8% and 97.7%, respectively).

## 5. Conclusions

The root canal anatomy of mandibular first molars in Black South Africans is diverse and complex. Differences were noted in the number of root canals across males, females, arch sides, and ages, although they were found to be statistically non-significant. The MM canal was identified in several molars of both males and females. No molars contained more than five canals. Patent ACs were more common in both M and D roots and in the apical region. Apical deltas were more common in males. Treating clinicians should take note of these findings, as they may reduce the risk of missed canals. The findings from this study are important, as Black South Africans make up roughly 79% of the South African population [46]. The findings of this study may help reduce the possibility of missed canals and contribute to the current knowledge base. Knowledge of accessory root canal anatomy is also important in endodontic management due to the potential portal of communication between the pulp and external root surface.

## Figures and Tables

**Table 1:** The number of canals in mandibular first molars in females and males, as well as on the left and right sides.

Canals	Male (*n* = 48)	Female (*n* = 38)	Total (*n* = 86)	Left (*n* = 42)	Right (*n* = 44)	Total (*n* = 86)
Two	1 (2.08%)	5 (13.16%)	6 (6.98%)	5 (11.90%)	1 (2.27%)	6 (6.98%)
Three	31 (64.58%)	17 (44.74%)	48 (55.81%)	21 (50.0%)	27 (61.36%)	48 (55.81%)
Four	10 (20.83%)	11 (28.95%)	21 (24.42%)	10 (23.81%)	11 (25.0%)	21 (24.42%)
Five	6 (12.5%)	5 (13.16%)	11 (12.79%)	6 (14.29%)	5 (11.36%)	11 (12.79%)
Total	48 (100%)	38 (100%)	86 (100%)	42 (100%)	44 (100%)	86 (100%)

**Table 2:** The number of canals in M and D roots.

Root	Canals	Male (*n* = 48)	Female (*n* = 38)	Total (*n* = 86)	Left (*n* = 42)	Right (*n* = 44)	Total (*n* = 86)
M							
	One	1 (2.08%)	5 (13.16%)	6 (6.98%)	5 (11.90%)	1 (2.27%)	6 (6.98%)
	Two	40 (83.33%)	24 (63.16%)	64 (74.12%)	31 (73.81%)	33 (75.0%)	64 (74.42%)
	Three	7 (14.58%)	9 (23.68%)	16 (18.60%)	6 (16.67%)	10 (22.73%)	16 (18.60%)
	Total	48 (100%)	38 (100%)	86 (100%)	42 (100%)	44 (100%)	86 (100%)
D							
	One	35 (72.92%)	26 (68.42%)	61 (70.93%)	29 (69.05%)	32 (72.73%)	61 (70.93%)
	Two	11 (22.92%)	11 (28.95%)	22 (25.58%)	10 (23.81%)	12 (27.27%)	22 (25.58%)
	Three	2 (4.17%)	1 (2.63%)	3 (3.49%)	3 (7.14%)	-	3 (3.49%)
	Total	48 (100%)	38 (100%)	86 (100%)	42 (100%)	44 (100%)	86 (100%)

M: mesial root; D: distal root.

**Table 3:** Number and frequency of accessory canals in the M root, including regions, types, and deltas.

		Male (*n* = 48)	Female (*n* = 38)	Total (*n* = 86)	Left (*n* = 42)	Right (*n* = 44)	Total (*n* = 86)
M Root							
	* AC	38 (79.17%)	27 (71.05%)	65 (75.58%)	32 (76.19%)	33 (75%)	65 (75.58%)
	Region						
	Coronal	7 (14.58%)	1 (2.63%)	8 (9.3%)	6 (14.29%)	2 (4.55%)	8 (9.3%)
	Mid-root	7 (14.58%)	2 (5.26%)	9 (10.47%)	4 (9.52%)	5 (11.36%)	9 (10.47%)
	Apical	35 (72.91%)	25 (65.79%)	60 (69.77%)	29 (69.05%)	31 (70.45%)	60 (69.77%)
	AC type or delta						
	Loops	5 (10.42%)	2 (5.26%)	7 (8.14%)	4 (9.52%)	3 (6.82%)	7 (8.14%)
	Blind	12 (25.0%)	6 (15.79%)	18 (20.93%)	9 (21.43%)	9 (20.45%)	18 (20.93%)
	Patent	29 (60.42%	26 (68.42%)	55 (63.95%)	27 (64.29%)	28 (63.67%)	55 (63.95%)
	Delta	11 (22.91%)	2 (5.26%)	13 (15.12%)	7 (16.67%)	6 (13.64%)	13 (15.12%)
	֍ AC						
	1	15 (31.25%)	14 (36.84%)	29 (33.72%)	11 (26.19%)	18 (40.91%)	29 (33.72%)
	2	10 (20.83%)	10 (26.32%)	20 (23.26%)	9 (21.43%)	11 (25%)	20 (23.26%)
	3	4 (8.33%)	3 (7.89%)	7 (8.14%)	4 (9.52%)	3 (6.82%)	7 (8.14%)
	4	3 (6.25%)	-	3 (3.49%)	2 (4.76%)	1 (2.27%)	3 (3.49%)
	5	1 (2.08%)	-	1 (1.16%)	1 (2.38%)	-	1 (1.16%)
	6	1 (2.08%)	-	1 (1.16%)	1 (2.38%)	-	1 (1.16%)
	Total	70 (70.82%)	43 (76.31%)	113 (71.37%)	60 (66.66%)	53 (75.00%)	113 (71.37%)

* Number of teeth where ACs are present; ֍ number of ACs excluding deltas.

**Table 4:** Number and frequency of accessory canals in the D root, including regions, types, and deltas.

		Male (*n* = 48)	Female (*n* = 38)	Total (*n* = 86)	Left (*n* = 42)	Right (*n* = 44)	Total (*n* = 86)
D Root							
	* AC	41 (85.42%)	24 (63.16%)	65 (75.58%)	32 (76.19%)	33 (75.00%)	65 (75.58%)
	Region						
	Coronal	1 (2.08%)	4 (10.53%)	5 (5.81%)	2 (4.76%)	3 (6.82%)	5 (5.81%)
	Mid-root	5 (10.42%)	5 (13.16%)	10 (11.63%)	5 (11.9%)	5 (11.36%)	10 (11.63%)
	Apical	41 (85.42%)	25 (65.79%)	66 (76.74%)	32 (76.19%)	34 (77.27%)	66 (76.74%)
	AC type or delta						
	Loops	4 (8.33%)	4 (10.53%)	8 (9.3%)	4 (9.52%)	4 (9.09%)	8 (9.3%)
	Blind	5 (10.42%)	9 (23.68%)	14 (16.28%)	7 (16.67%)	7 (15.91%)	14 (16.28%)
	Patent	29 (60.42%	17 (44.74%)	46 (53.49%)	20 (47.62%)	26 (59.09%)	46 (53.49%)
	Delta	13 (27.08%)	5 (13.16%)	18 (20.93%)	12 (28.57%)	6 (13.64%)	18 (20.93%)
	֍ AC						
	1	15 (31.25%)	8 (21.05%)	23 (26.74%)	11 (26.19%)	12 (27.27%)	23 (26.74%)
	2	12 (25.0%)	9 (23.68%)	21 (24.42%)	8 (19.05%)	13 (29.55%)	21 (24.42%)
	3	3 (6.25%)	3 (7.89%)	6 (6.98%)	2 (4.76%)	4 (9.09%)	6 (6.98%)
	4	1 (2.08%)	1 (2.63%)	2 (2.33%)	2 (4.76%)	-	2 (2.33%)
	Total	52 (64.58%)	39 (55.25%)	91 (61.64%)	41 (54.76%)	50 (65.91%)	91 (60.47%)

* Number of teeth where ACs are present; ֍ number of ACs excluding deltas.

## Data Availability

The data presented in this study are available from the corresponding author upon request. The data are not publicly available due to ethical reasons.

## Abbreviations

The following abbreviations are used in this manuscript:

M	Mesial
D	Distal
MM	Middle-mesial
MD	Middle-distal
AC	Accessory canal
ACs	Accessory canals
Micro-CT	Micro-computed tomography
STROBE	Strengthening the reporting of observational studies in epidemiology
3D	Three-dimensional
CEJ	Cemento–enamel junction
L	Left
R	Right
